# Exogenous N-Acetyl-L-cysteine Improves Soybean Saline–Alkali Tolerance by Enhancing the hGSH Pathway to Alleviate Oxidative Damage

**DOI:** 10.3390/plants15162445

**Published:** 2026-08-11

**Authors:** Suyu Chen, Wei Chen, Xin Li, Wenshuo Zhou, Ran Sun, Lingyu Zhou, Yuxian Zhang, Qiang Zhao

**Affiliations:** 1 Key Laboratory of Ministry of Agriculture and Rural Affairs of Soybean Mechanized Production, Heilongjiang Bayi Agricultural University, Daqing 163319, China; chensuyu3310@163.com (S.C.); 13039780502@163.com (W.C.); 13936979283@163.com (X.L.); 18746570490@136.com (W.Z.); srr2674792279@163.com (R.S.); 13836919783@163.com (L.Z.); 2 National Coarse Cereals Engineering Research Center, Daqing 163319, China

**Keywords:** reactive oxygen species, AsA–(h)GSH cycle, oxidative DNA damage, cellular ultrastructure, genotype-specific response

## Abstract

Soil salinization and alkalization severely threaten global agricultural sustainability. Although N-acetylcysteine (NAC) is well known as an antioxidant in animal research, its effects on plant salt–alkali tolerance and the underlying mechanisms remain unclear. This study investigated the regulatory effects of foliar-sprayed NAC on two soybean (*Glycine max*) cultivars—salt-tolerant HF50 and salt-sensitive HN95—under saline–alkali stress. Exogenous NAC alleviated seedling growth inhibition by supplying cysteine, which elevated glutamate–cysteine ligase (GCL) activity and promoted homoglutathione (hGSH) accumulation. It further strengthened the ascorbic acid (AsA)–glutathione/homoglutathione ((h)GSH) cycle and elevated antioxidant enzyme activities, thereby relieving oxidative injury, while also activating DNA repair pathways to preserve genomic stability and protecting chloroplast and mitochondrial integrity. Notably, distinct genotype- and tissue-specific responses to NAC were detected. HN95 relied primarily on root antioxidant defenses in a strongly concentration-dependent manner, whereas HF50 coordinated antioxidant metabolism and DNA repair more effectively, showing consistent responses to NAC concentrations. These findings elucidate the coordinated physiological and molecular mechanisms by which exogenous NAC mitigates saline–alkali damage in soybean, providing a theoretical foundation for using NAC to enhance crop stress resilience and develop sustainable cultivation techniques for saline–alkali soils.

## 1. Introduction

Soil salinization, driven by both natural environmental variations and anthropogenic activities such as improper irrigation and excessive chemical fertilization, is a widespread global issue that severely constrains agriculture productivity [1]. Saline–alkali stress triggers osmotic stress, ion toxicity and oxidative damage in plants, disrupting physiological and metabolic homeostasis. This disruption suppresses photosynthesis, water uptake and assimilate transport, leading to reduced biomass, stunted growth and even plant mortality [2]. These adverse consequences lead to vegetation degradation, constrain the geographical distribution and yield of crops, and threaten food security and ecosystem stability [3,4]. Against this backdrop, climate change, rising food demand, and population growth have made enhancing crop stress tolerance a prerequisite for sustaining agricultural productivity in the coming decades [5]. Accordingly, elucidating the mechanisms of plant salt–alkali tolerant and developing effective cultivation practices have become critical research priorities in plant science and agronomy.

Under saline–alkali stress, excessive reactive oxygen species (ROS) accumulate in plant cells and oxidize membrane lipids, functional proteins, and nucleic acids, leading to lipid peroxidation (LPO), protein inactivation, and oxidative DNA damage [6]. The guanine oxidation product 8-hydroxy-2′-deoxyguanosine (8-OHdG) is a well-established biomarker of oxidative DNA damage [7]. Previous studies have verified that saline–alkali stress significantly elevates endogenous 8-OHdG concentrations and impairs genomic stability in plants [8]. Unrepaired oxidative DNA damage disrupts DNA replication and transcription, and can trigger programmed cell death under severe stress [9,10].

To counteract ROS-derived oxidative toxicity, plants have evolved a ROS scavenging system comprising enzymatic and non-enzymatic antioxidants to sustain intracellular redox homeostasis. Within the enzymatic antioxidant system, superoxide dismutase (SOD) first converts superoxide anions into hydrogen peroxide, which is subsequently broken down into H_2_O and O_2_ via catalysis by peroxidase (POD), catalase (CAT) and ascorbate peroxidase (APX) [11]. The non-enzymatic antioxidant pool mainly comprises ascorbic acid (AsA), glutathione (GSH), flavonoids, carotenoids and tocopherols; these compounds either directly quench ROS or stabilize the intracellular environment through redox cycling [12]. Among these antioxidant components, GSH establishes an efficient redox regulatory network though the AsA-GSH cycle and serves as a core metabolite mitigating oxidative damage [13].

The AsA–GSH cycle operates in chloroplasts, mitochondria, peroxisomes, and the cytoplasm, where it mediates ROS scavenging and antioxidant regeneration through a series of enzymatic reactions [14]. In this pathway, APX catalyzes the reduction of H_2_O_2_ using AsA as a substrate to generate monodehydroascorbate (MDHA). MDHA can be reduced back to AsA by monodehydroascorbate reductase (MDHAR) or undergo spontaneous dismutation to yield AsA and dehydroascorbic acid (DHA). DHA is subsequently recycled to AsA by dehydroascorbate reductase (DHAR) at the expense of GSH, while GSH is simultaneously oxidized to oxidized glutathione (GSSG). Finally, glutathione reductase (GR) catalyzes the reduction of GSSG back to GSH using NADPH as the electron donor, thereby completing the cycle [15]. Numerous studies have confirmed that exogenous regulators such as methyl jasmonate, melatonin, and sulfonated graphene nanomaterials can enhance the activities of key enzymes including APX, DHAR and GR, thereby promoting sustained regeneration of AsA and GSH and markedly improving plant tolerance to various abiotic stresses, such as low temperature combined with low light, saline–alkali, and nitrogen deficiency stress [16,17,18]. Collectively, the AsA–GSH cycle serves as a crucial target through which exogenous compounds modulate plant stress tolerance.

Notably, homoglutathione (hGSH) functions as the predominant thiol compound supporting cellular redox balance and oxidative stress buffering in *Papilionoideae* legumes [19,20]. Both hGSH and GSH are synthesized from the common precursor γ-glutamylcysteine (γ-EC) generated during the rate-limiting step; in the final step, hGSH synthase (hGSHS) incorporates β-alanine rather than glycine [19,20,21]. Despite this difference at the C-terminal amino acid, hGSH functions via the conserved cysteinyl thiol group [14]. Studies have demonstrated that DHAR and GR from legumes efficiently use both hGSH and its oxidized form, oxidized homoglutathione (hGSSG) as substrates, with catalytic efficiencies comparable to those for GSH [22]. Accordingly, the hGSH-mediated AsA–(h)GSH cycle represents a core target for elucidating how exogenous compounds regulate stress tolerance in soybean.

N-acetyl-L-cysteine (NAC) is an acetylated derivative of L-cysteine and has been widely used in clinical practice as a mucolytic agent and an antidote against acetaminophen poisoning [23]. In mammalian systems, NAC mainly acts as a cysteine precursor to facilitate GSH biosynthesis and strengthen cellular antioxidant capacity. In addition to its direct radical-scavenging activity conferred by the thiol group, NAC modulates redox signaling by generating reactive sulfur species, including sulfane sulfur and hydrogen sulfide [24,25,26,27]. These properties, together with its greater stability compared with free L-cysteine, make NAC a versatile antioxidant agent [28]. 

In plants, exogenous NAC alleviates oxidative damage caused by diverse abiotic stresses through replenishing endogenous GSH pools, sustaining the ascorbate–glutathione cycle, and enhancing the activities of antioxidant enzymes such as SOD, CAT, and POD [29,30,31,32]. Saline–alkali stress typically induces a severe oxidative burst and perturbs GSH homeostasis in plant tissues, suggesting that the glutathione-dependent antioxidant system may be a key target for stress mitigation [33]. Our previous study showed that 1 mmol·L^−1^ NAC enhanced antioxidant enzyme activities and photosynthetic capacity in the tolerant genotype HF50 under saline–alkali stress, an effect associated with the alleviation of growth inhibition [34]. However, several key questions remain unresolved following this single-concentration, single-genotype study: whether elevated NAC concentrations trigger enhanced or distinct protective responses; whether these regulatory pathways vary between soybean cultivars with contrasting saline–alkali tolerance; and what physiological mechanisms govern these responses, particularly regarding homoglutathione (hGSH) metabolism and DNA damage and repair.

To address these questions, two soybean cultivars with contrasting saline–alkali tolerance—HF50 (tolerant) and HN95 (sensitive)—were treated with 1, 2, and 3 mmol·L^−1^ NAC. By systematically comparing their physiological responses, we investigated the dose-dependent and genotype-specific mechanisms of NAC-mediated saline–alkali tolerance in soybean from four dimensions: (1) plant growth performance, (2) glutathione metabolism, (3) antioxidant defense regulation, and (4) DNA damage and repair. This study provides a theoretical foundation for developing stress-resistant cultivation strategies for soybean in saline–alkali soils.

## 2. Results

### 2.1. Effects of Exogenous NAC on the Growth of Soybean Seedlings Under Saline–Alkali Stress

**Figure 1:**
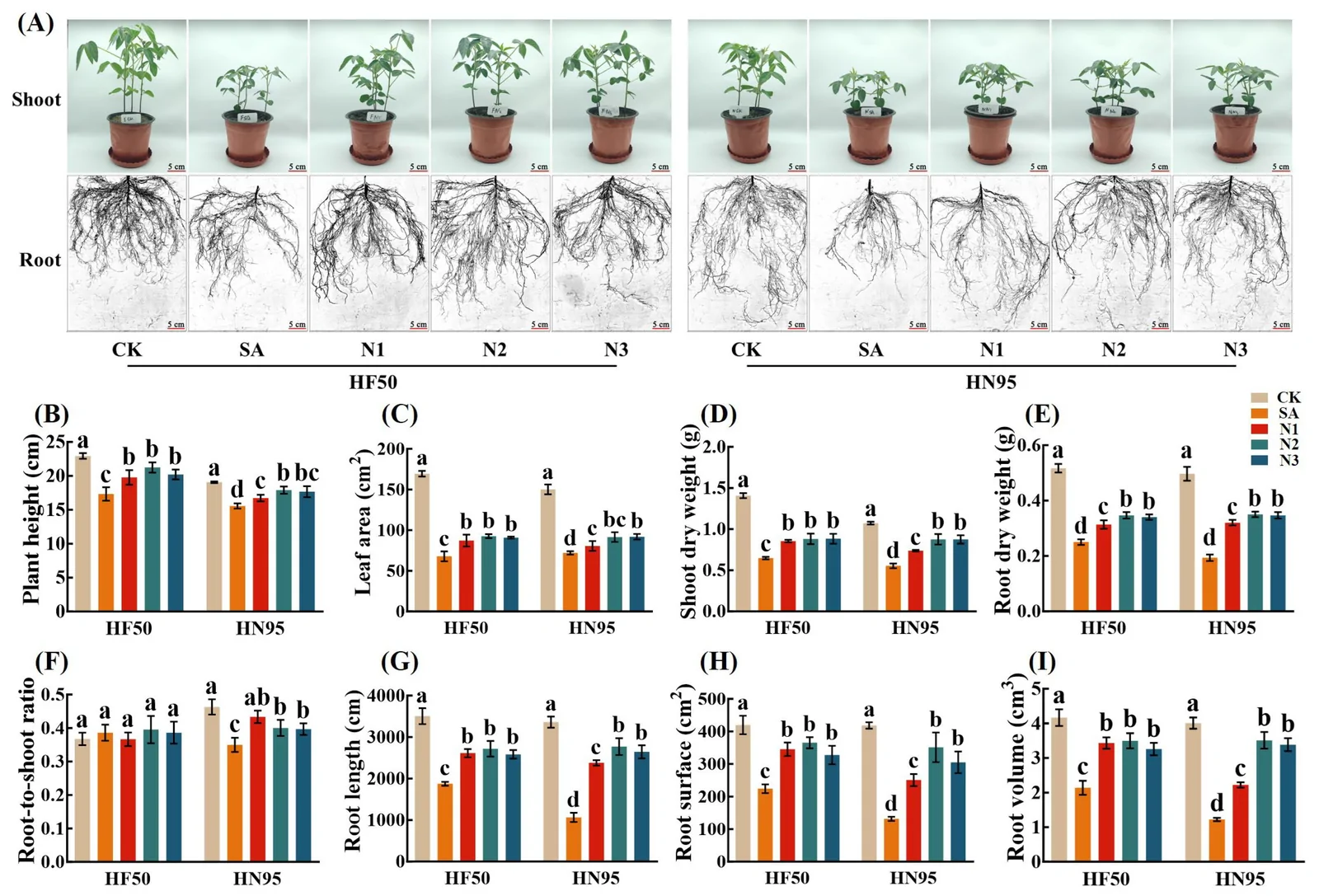
Exogenous NAC alleviates the growth inhibition of soybean seedlings under saline–alkali stress. (**A**) Shoot and root phenotypic characteristics of HF50 and HN95 soybean seedlings, representing salt-tolerant and salt-sensitive cultivars, respectively, after 25 days of continuous culture under saline–alkali stress with NAC treatments. (**B**–**I**) Quantitative analysis of seedling growth indicators, including plant height (**B**), leaf area (**C**), shoot dry weight (**D**), root dry weight (**E**), root-to-shoot ratio (R/S, root dry weight/shoot dry weight) (**F**), root length (**G**), root surface area (**H**), and root volume (**I**). Treatments comprised a control (CK) group, a single saline–alkali (SA) stress group, and saline–alkali stress groups supplemented with 1, 2, and 3 mmol·L^−1^ N-acetyl-L-cysteine (NAC), designated N1, N2 and N3, respectively. Data are mean ± SD of three independent biological replicates. Different lowercase letters indicate significant differences at *p* < 0.05.

To clarify the effects of NAC treatment on the growth of soybean plants under saline–alkali stress, morphological parameters and biomass of soybean cultivars HF50 and HN95 were measured after 25 days of treatment with different concentrations of NAC. As shown in Figure 1, saline–alkali stress (SA) significantly reduced plant height, leaf area, root length, root surface area, root volume, root tip number and biomass in both HF50 and HN95 when compared with the control (CK). For the salt-tolerant cultivar HF50, the reduction rates of plant height, leaf area and shoot dry weight (24.42%, 59.94% and 53.93%, respectively) were higher than those of the salt-sensitive cultivar HN95 (18.36%, 52.07% and 48.42%, respectively). In contrast, HF50 exhibited lower reduction rates in root dry weight, root length, root surface area and root volume (51.61%, 46.54%, 46.60% and 48.71%, respectively) than HN95 (61.07%, 68.33%, 68.56% and 69.49%, respectively). In addition, saline–alkali stress significantly decreased the root-to-shoot ratio (R/S) only in HN95.

Compared with saline–alkali stress alone, exogenous NAC markedly alleviated growth inhibition in both cultivars and significantly increased plant height, leaf area, biomass, root length, root surface area and root volume. Under different NAC concentrations, all morphological and biomass indicators of HF50 showed similar increasing amplitudes, whereas HN95 displayed distinct concentration-dependent responses. The increments of all indicators under N2 and N3 treatments were significantly greater than those under N1 treatment, indicating an obvious dose-dependent effect. Exogenous NAC exerted no significant influence on the R/S ratio of HF50, but significantly elevated the R/S value of HN95 (Figure 1).

### 2.2. Effects of Exogenous NAC on hGSH Biosynthesis in Soybean Seedlings Under Saline–Alkali Stress

To investigate the regulatory effects of NAC on the hGSH biosynthetic pathway in soybean under saline–alkali stress, the activities of rate-limiting enzymes and transcript abundance of related functional genes were determined in the leaves and roots of two cultivars after 2 days of foliar spraying with NAC. The Results showed that, compared with the control, saline–alkali stress significantly increased cysteine concentration in the roots of HF50, but not in the roots of HN95 or in the leaves of either cultivar. In contrast, saline–alkali stress markedly elevated GCL activity in the leaves of both cultivars, whereas root GCL activity was significantly suppressed. qRT-PCR analysis revealed that saline–alkali stress significantly induced the expression of *GmGSH1*, *GmHGS*, *GmSERAT1*, and *GmSERAT2* in the leaves of both cultivars. In HF50 roots, *GmHGS*, *GmSERAT1*, and *GmSERAT2* were significantly upregulated, whereas *GmGSH1* was significantly repressed; in HN95 roots, only *GmHGS* transcript abundance was notably elevated.

**Figure 2:**
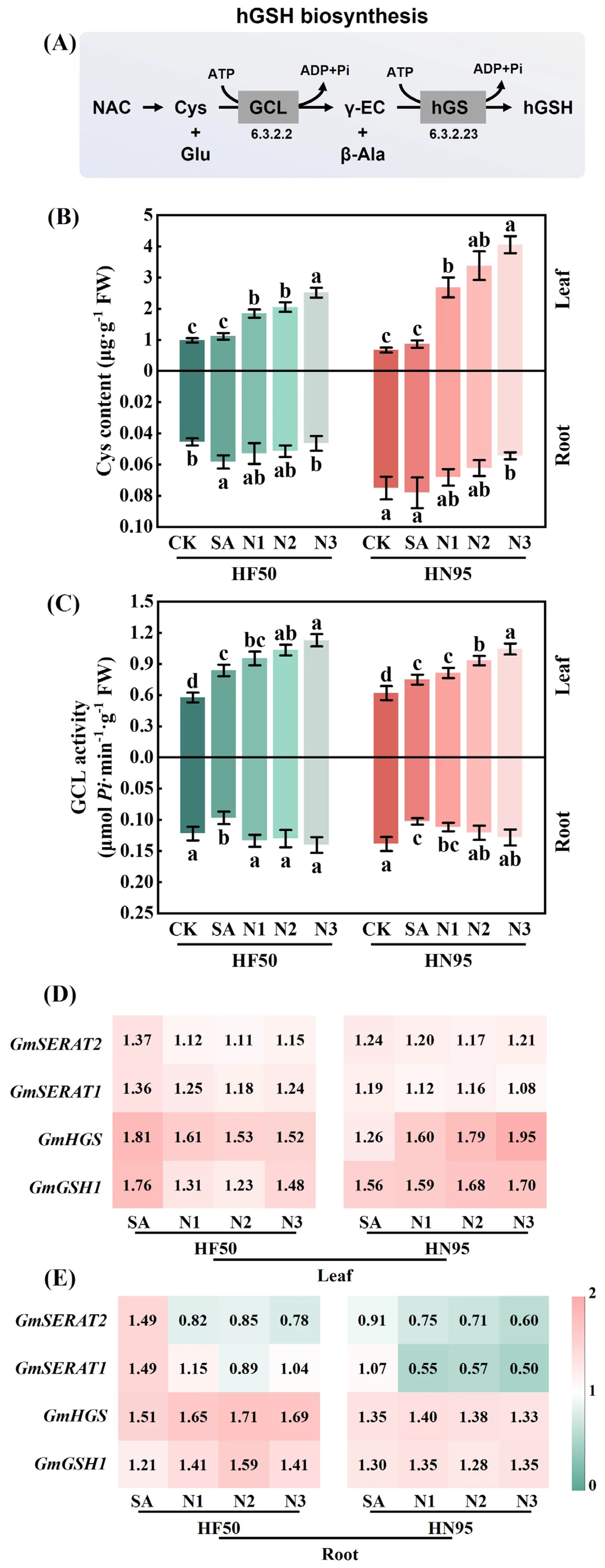
Exogenous NAC regulates hGSH biosynthesis in soybean seedlings under saline–alkali stress. (**A**) Schematic diagram of hGSH biosynthetic pathway. (**B**) Cysteine content. (**C**) γ-glutamylcysteine ligase (GCL) activity. (**D**,**E**) The heatmap visualizes the expression levels of hGSH biosynthetic genes under saline–alkali stress and NAC treatment, including genes encoding GCL (*GmGSH1*), hGSH synthase (*GmHGS*), and serine acetyltransferases (*GmSERAT1* and *GmSERAT2*). The expression level of the control group was defined as 1. The color gradient represents relative expression levels, with upregulation shown in red and downregulation in green. Data are mean ± SD of three independent biological replicates. Different lowercase letters indicate significant differences at *p* < 0.05.

Compared with saline–alkali stress alone, exogenous NAC significantly increased cysteine content in the leaves of both cultivars in a concentration-dependent manner, with HN95 showing a more pronounced response than HF50. In roots, NAC reduced cysteine content, although this decrease reached statistical significance only at the N3 concentration. Additionally, exogenous NAC also significantly enhanced GCL activity in both leaves and roots of the two cultivars, and leaf GCL activity exhibited a clear concentration-dependent pattern. qRT-PCR analysis revealed that exogenous NAC markedly increased transcript abundance of *GmGSH1*, *GmHGS*, *GmSERAT1* and *GmSERAT2* in the leaves of both cultivars relative to saline–alkali stress alone. In HF50 roots, *GmHGS*, *GmSERAT1*, and *GmSERAT2* were upregulated by NAC, whereas *GmGSH1* was significantly downregulated; in HN95 roots, only *GmHGS* expression was significantly induced (Figure 2).

### 2.3. Effects of Exogenous NAC on AsA–(h)GSH Cycle and Antioxidant Enzyme System in Soybean Seedlings Under Saline–Alkali Stress

To investigate the antioxidant mechanism by which NAC alleviates saline–alkali stress in soybean, the contents of AsA–(h)GSH cycle metabolites and the activities of corresponding antioxidant enzymes were determined in the leaves and roots of two cultivars after 2 days of foliar spraying with NAC. Compared with the control, saline–alkali stress did not alter (h)GSH concentration in leaves or roots of either cultivar, but markedly elevated the contents of (h)GSSG, AsA, and DHA in both tissues. In addition, saline–alkali stress also markedly reduced the activities of AsA-(h)GSH cycle-related enzymes in the leaves and roots of both cultivars, as well as SOD, CAT and APX activities in HN95 roots. This redox shift was accompanied by a decrease in both the (h)GSH/(h)GSSG and AsA/DHA ratios across tissues and cultivars (Figure S1). Conversely, the activities of SOD, CAT, APX and POD were significantly elevated in leaves of both cultivars and roots of HF50.

**Figure 3:**
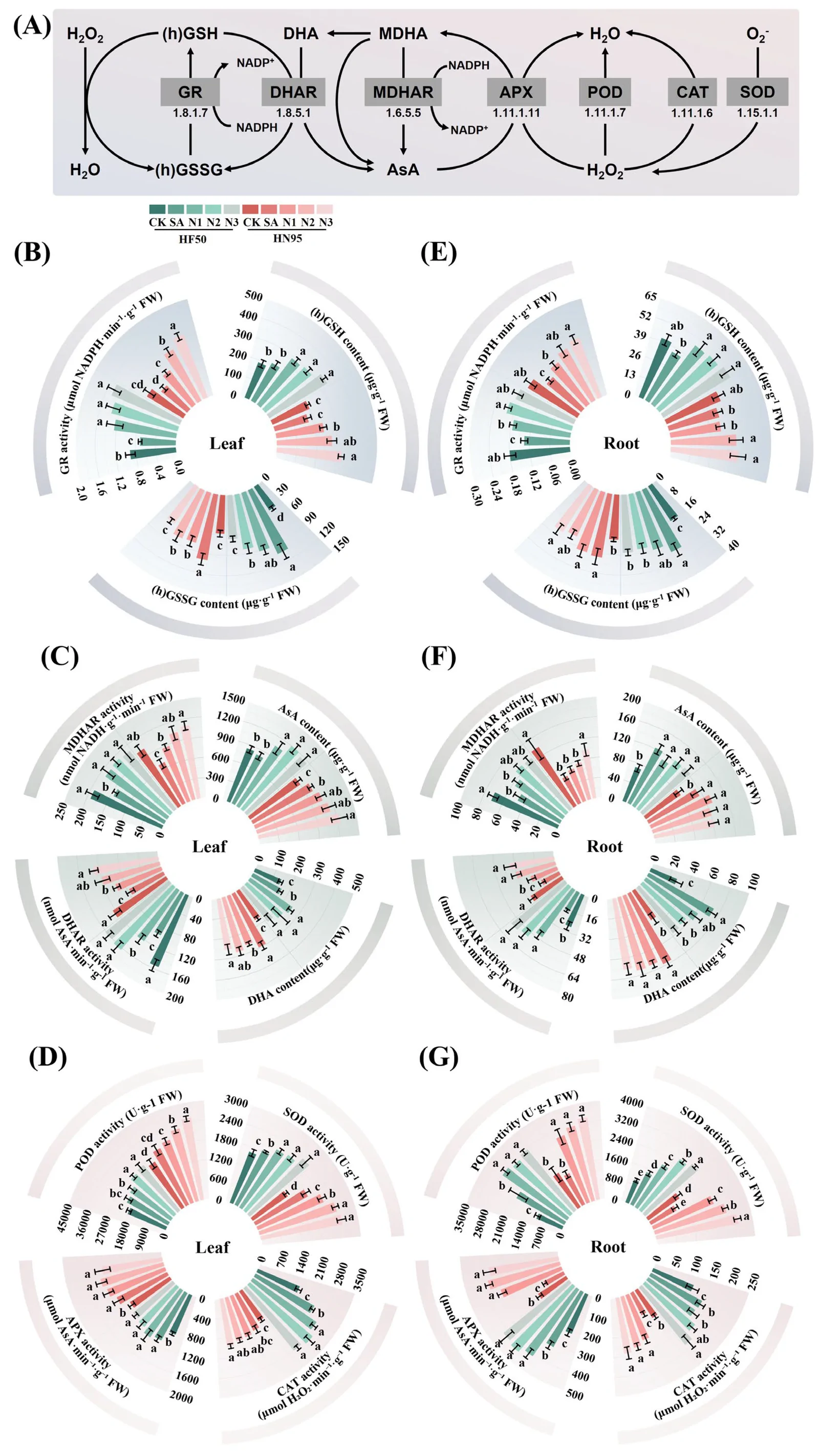
Exogenous NAC enhances AsA–(h)GSH cycle operation and antioxidant enzyme activities in soybean seedlings under saline–alkali stress. (**A**) Schematic overview of the AsA–(h)GSH cycle and major ROS scavenging enzymes. (**B**–**D**) Parameters in leaves: (**B**) (h)GSH and (h)GSSG contents, GR activity; (**C**) AsA and DHA contents, DHAR and MDHAR activities; (**D**) SOD, CAT, APX and POD activities. (**E**–**G**) Parameters in roots: same as (**B**–**D**). Data are mean ± SD of three independent biological replicates. Different lowercase letters indicate significant differences at *p* < 0.05.

Relative to the single saline–alkali stress treatment, exogenous NAC significantly increased (h)GSH levels in the leaves and roots of HF50 and HN95, with a clear concentration-dependent elevation observed in HN95. Exogenous NAC also raised leaf AsA concentrations in both cultivars and specifically increased leaf DHA content only in HF50. Meanwhile, NAC significantly reduced (h)GSSG contents in the leaves of both cultivars and the roots of HF50; DHA levels were only lowered significantly in HF50 roots, whereas root AsA concentrations in both cultivars and root DHA levels in HN95 remained unaffected. In addition, NAC remarkably upregulated the activities of GR, DHAR, MDHAR and major ROS scavenging enzymes (SOD, CAT, APX, POD) in all leaf and root samples. The activation of GR, DHAR, MDHAR, SOD and POD exhibited obvious concentration dependence in HN95, whereas HF50 displayed greater increases in the activities of several enzymes such as GR, DHAR and CAT than HN95 (Figure 3). In line with this coordinated restoration of the reductive state, NAC substantially elevated the (h)GSH/(h)GSSG and AsA/DHA ratios in the leaves and roots of both cultivars (Figure S1).

### 2.4. Effects of Exogenous NAC on ROS Accumulation and LPO Peroxidation in Soybean Seedlings Under Saline–Alkali Stress

To explore the effects of NAC on oxidative stress in soybean seedlings under saline–alkali stress, the accumulation of ROS and the levels of LPO and MDA content were measured in leaves and roots of two cultivars after 2 days of foliar spraying with NAC. The results showed that compared with the control group, saline–alkali stress significantly increased the contents of O_2_^−^ and H_2_O_2_, along with the staining intensity and distribution area of Nitro-Blue Tetrazolium (NBT) and 3,3′-Diaminobenzidine (DAB) in leaves and roots of HF50 and HN95 seedlings. The levels of LPO and malondialdehyde (MDA) also rose remarkably. Specifically, the increment magnitudes of O_2_^−^, H_2_O_2_ and MDA in HF50 leaves were higher than those in HN95, whereas the increases in these indicators in HF50 roots were lower compared with HN95.

**Figure 4:**
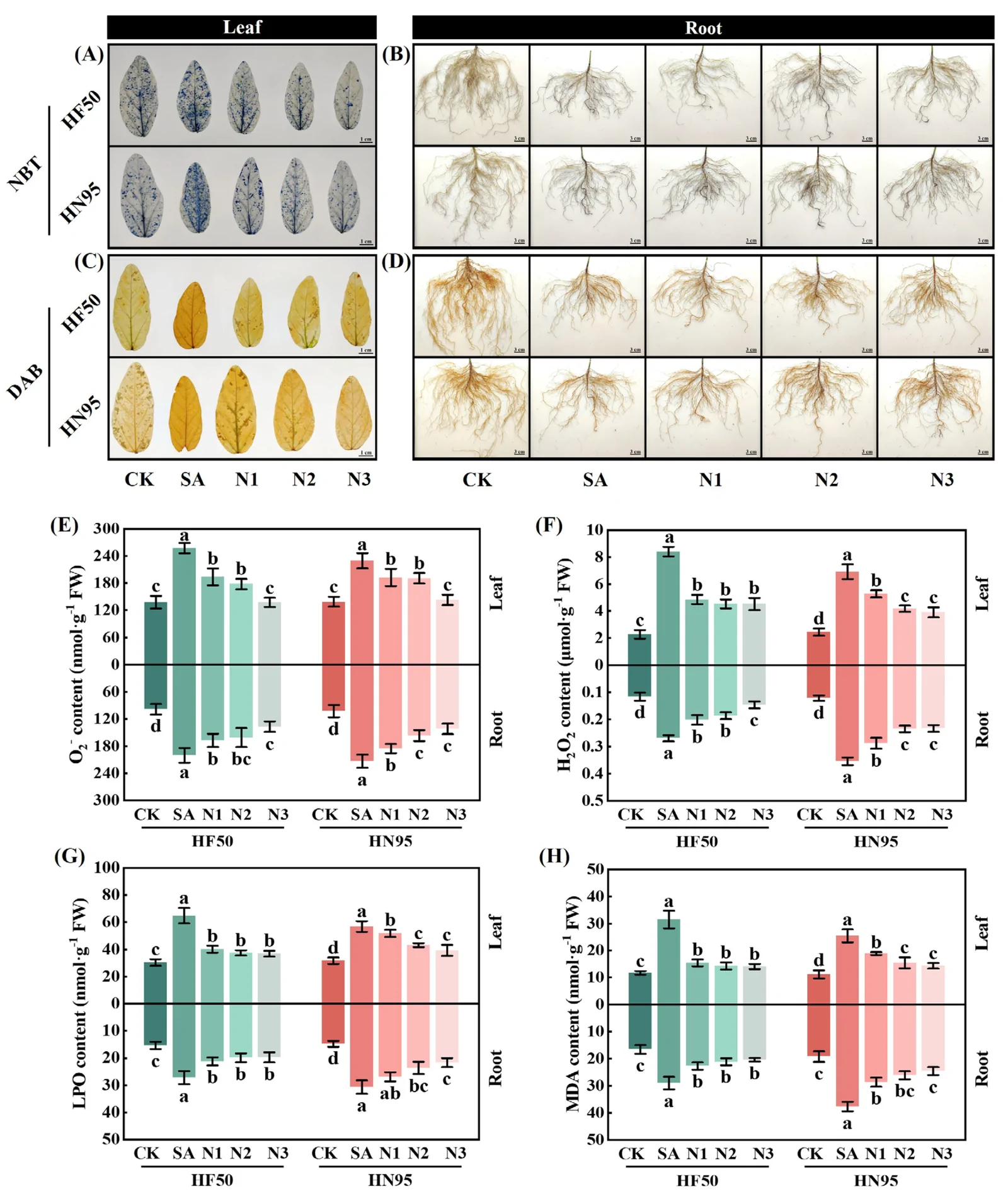
Exogenous NAC alleviates ROS accumulation and LPO in soybean seedlings under saline–alkali stress. NBT staining for O_2_^−^ in leaves (**A**) and roots (**B**). DAB staining for H_2_O_2_ in leaves (**C**) and roots (**D**). Quantification of O_2_^−^ content (**E**), H_2_O_2_ content (**F**), LPO content (**G**), and MDA content (**H**) in leaves and roots of HF50 and HN95. Data are mean ± SD of three independent biological replicates. Different lowercase letters indicate significant differences at *p* < 0.05.

Compared with the sole saline–alkali treatment, exogenous NAC application substantially reduced the accumulation and stained area of O_2_^−^ and H_2_O_2_, and lowered LPO and MDA contents in leaves and roots of both cultivars under saline–alkali stress. The reduction extents of all indicators under N2 and N3 treatments were greater than those under N1, exhibiting an obvious dose-dependent effect (Figure 4). To put these NAC-induced reductions into a quantitative integrative perspective, recovery rates—calculated as the proportion of saline–alkali-stress-induced damage offset by NAC—were computed for O_2_^−^, H_2_O_2_, LPO and MDA. NAC markedly elevated these recovery rates in the leaves and roots of both HF50 and HN95, with higher magnitudes at N2 and N3 than at N1, mirroring the dose-responsive trend noted above (Figure S2).

### 2.5. Effects of Exogenous NAC on DNA Damage and Genome Stability in Soybean Seedlings Under Saline–Alkaline Stress

To further reveal the regulatory role of exogenous NAC in DNA damage and oxidative DNA damage under saline–alkali stress, the DNA polymorphism and 8-OHdG content were determined in the leaves and roots of two cultivars after 2 days of foliar spraying with NAC. The results showed that compared with the control group, saline–alkali stress significantly increased the 8-OHdG contents in leaves and roots of both cultivars. This stress only induced obvious Random Amplified Polymorphic DNA (RAPD) polymorphic variations in root genomic DNA and reduced root genome stability, while exerting no significant effects on leaf genomic DNA. Among the two cultivars, HF50 exhibited fewer RAPD polymorphic bands in roots and possessed higher genome stability than HN95.

**Figure 5:**
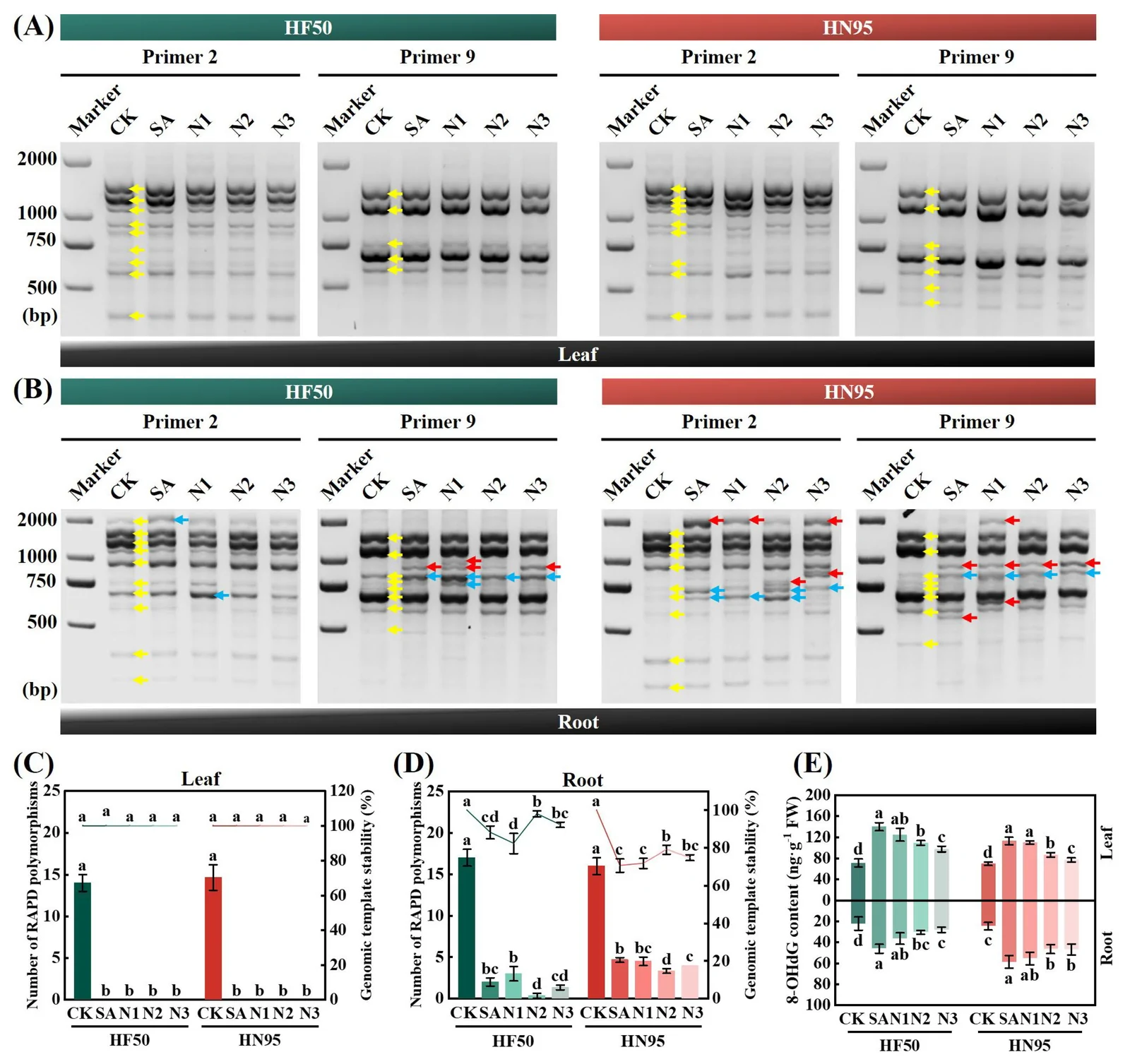
Exogenous NAC regulates DNA damage and genome stability in soybean seedlings under saline–alkali. RAPD profiles were amplified using random primers 2 and 9 in leaves (**A**) and roots (**B**) of soybean seedlings treated with various concentrations of exogenous NAC under saline–alkaline stress. Yellow arrows indicate characteristic bands in the control; red arrows show bands that appeared or disappeared relative to the control; blue arrows indicate bands with grayscale intensity changes greater than 50% compared with the control. Quantitative analysis of RAPD profiles and genomic template stability was conducted in leaf (**C**) and root (**D**) samples. The bar charts show the number of RAPD polymorphisms, while the line graphs indicate genomic template stability. CK refers to the total band number of the control group; SA and N1–N3 denote the count of variant bands compared with CK. (**E**) 8-OH-dG levels in leaves and roots. All data are expressed as mean ± SD from three independent replicates. Significant differences between treatments were determined by multiple comparisons (*p* < 0.05), as indicated by different lowercase letters.

Compared with the single saline–alkali stress treatment, exogenous NAC markedly decreased 8-OHdG concentrations in leaves and roots of both cultivars in a dose-dependent manner. In addition, exogenous NAC had no obvious influence on leaf genomic DNA under saline–alkali conditions, maintaining stable leaf genomes. Meanwhile, exogenous NAC significantly alleviated RAPD polymorphic variations of root genomic DNA and improved root genome stability (Figure 5). To put this NAC-induced reduction into a quantitative integrative perspective, the recovery rate—calculated as the proportion of saline–alkali-stress-induced DNA oxidation offset by NAC—was also computed for 8-OHdG. NAC markedly elevated the 8-OHdG recovery rate in the leaves and roots of both HF50 and HN95, with the percentage offset larger at N2 and N3 than at N1, in line with the dose-dependent fall in absolute 8-OHdG concentrations described above (Figure S2).

### 2.6. Effects of Exogenous NAC on the DNA Damage Repair System of Soybean Seedlings Under Saline–Alkali Stress

To explore the mechanism by which exogenous NAC relieves saline–alkali stress-induced DNA damage in soybean, the transcript levels of genes involved in diverse DNA repair pathways were determined in the leaves and roots of two cultivars after 2 days of foliar spraying with NAC. qRT-PCR analysis revealed that saline–alkali stress upregulated the expression of key DNA repair genes in the leaves of both cultivars but downregulated them in the roots. In addition, HF50 exhibited higher expression levels of 8-OHdG regulatory genes but lower expression of key homologous recombination (HR) pathway genes compared with HN95.

**Figure 6:**
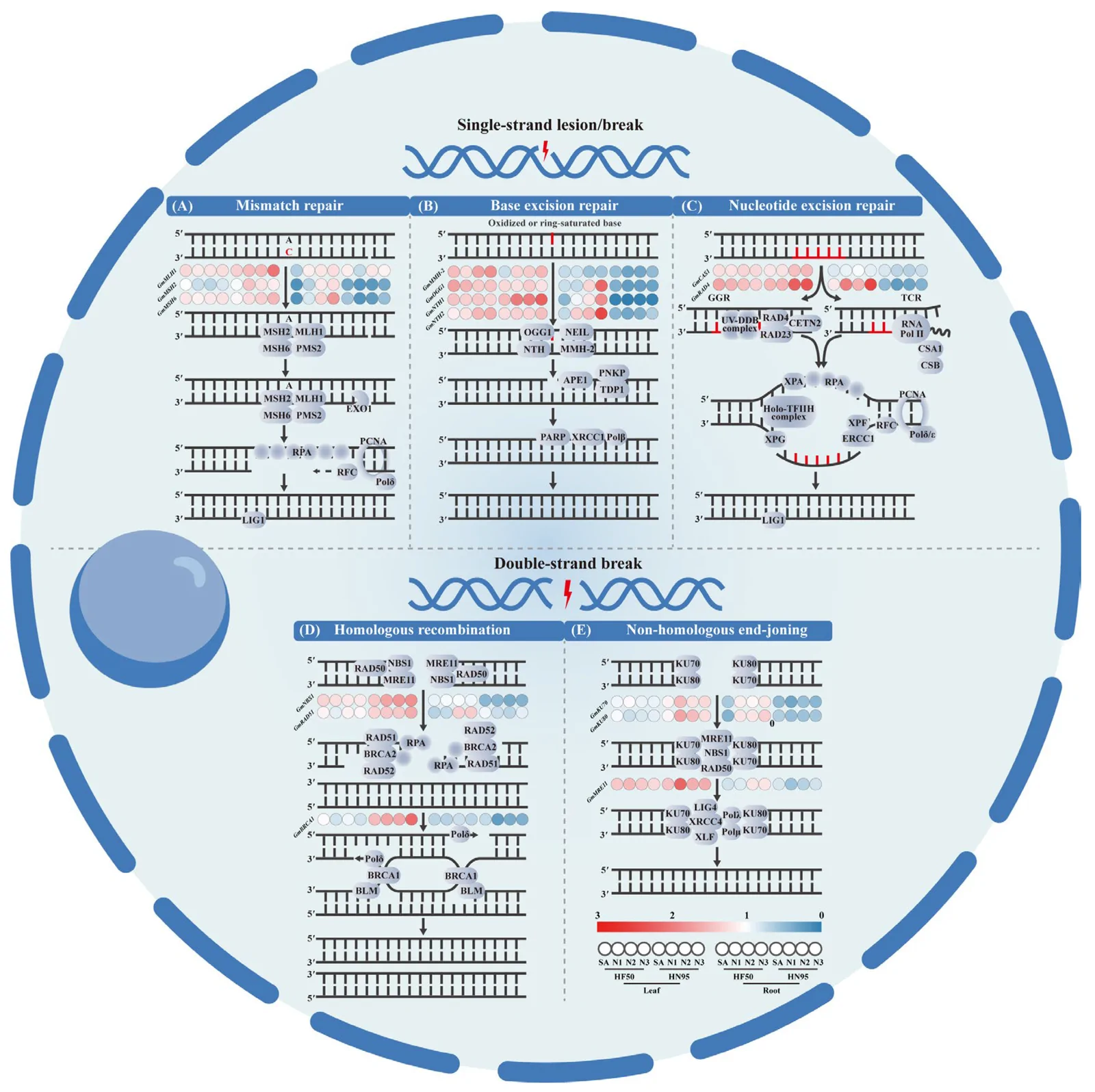
Regulation of exogenous NAC on the DNA damage repair system in roots and leaves of soybean seedlings under saline–alkali stress. The heatmap shows qRT-PCR-based expression of DNA damage repair-related genes. These genes cover five categories: (**A**) mismatch repair (MMR): *GmMSH2*, *GmMSH6*, *GmMLH1*; (**B**) base excision repair (BER): *GmMMH-2*, *GmOGG1*, *GmNTH1*, *GmNTH2*; (**C**) nucleotide excision repair (NER): *GmRAD4* (*XPC* homolog), *GmCSA1*; (**D**) homologous recombination (HR): *GmBRCA1*, *GmRAD51*, *GmNBS1*; (**E**) non-homologous end-joining (NHEJ): *GmKU70*, *GmKU80*, *GmMRE11*. The expression level of the control group was defined as 1. The color gradient represents relative expression levels (red: upregulation; blue: downregulation).

In leaves, compared with saline–alkali stress alone, exogenous NAC generally maintained the expression levels of DNA repair-related genes in HF50, with certain genes (e.g., *GmOGG1* and *GmNTH2*) showing clear concentration-dependent upregulation. In HN95 leaves, exogenous NAC overall promoted the expression of these genes, also in a concentration-dependent manner. In roots, exogenous NAC broadly upregulated DNA repair genes in HF50 in a concentration-dependent manner, with particularly prominent upregulation of *GmOGG1*, *GmRAD4*, *GmNTH1*, and *GmNTH2*. However, NAC failed to reverse the saline–alkali-induced downregulation of these genes in HN95 roots, merely maintaining their pre-existing low expression levels (Figure 6).

### 2.7. Effects of Exogenous NAC on Leaf Ultrastructure, Photosynthetic Rate and Root Activity of Soybean Under Saline–Alkali Stress

**Figure 7:**
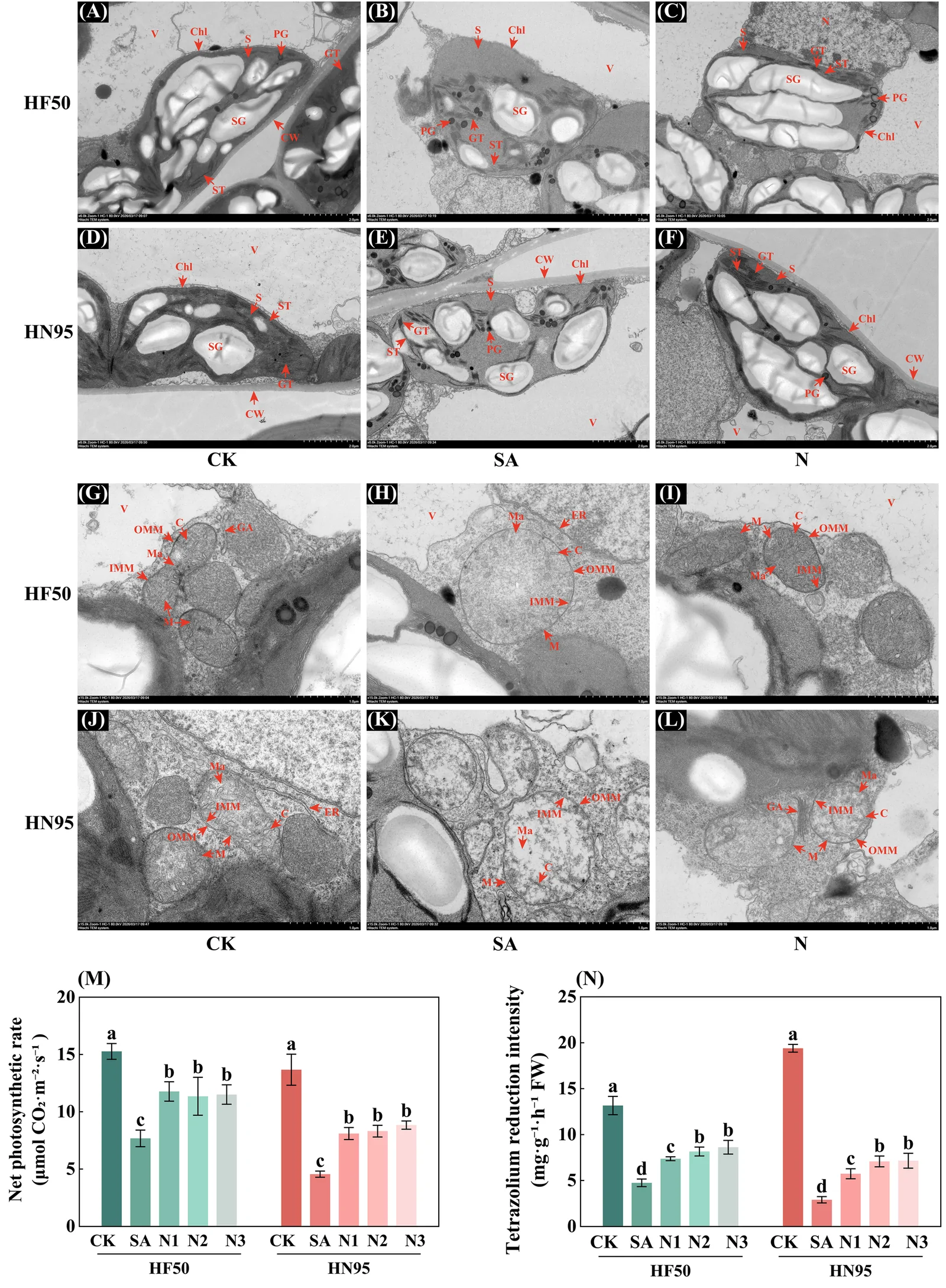
Alleviating effects of exogenous NAC on leaf ultrastructure, photosynthetic rate and root activity of soybean under saline–alkali stress. TEM micrographs displayed the leaf subcellular ultrastructure of soybean seedlings under three treatments: control (CK), saline–alkali stress (SA), and 2 mmol L^−1^ NAC treatment (N). Panels (**A**–**F**) show chloroplast ultrastructure at 6000-fold magnification; panels (**G**–**L**) show mitochondrial ultrastructure at 15,000-fold magnification. Panels (**M**,**N**) represent the net photosynthetic rate and tetrazolium reduction intensity (root activity) of soybean, respectively. Abbreviations in the figure: N, nucleus; CW, cell wall; V, vacuole; Chl, chloroplast; SG, starch grain; PG, plastoglobule; M, mitochondrion; GT, grana thylakoid; S, stroma; ST, stroma thylakoid; OMM, outer mitochondrial membrane; IMM, inner mitochondrial membrane; C, crista (the folded region of IMM); Ma, mitochondrial matrix; ER, endoplasmic reticulum; GA, Golgi apparatus. Data are mean ± SD of three independent biological replicates. Different lowercase letters indicate significant differences at *p* < 0.05.

To explore the alleviating effects of exogenous NAC on physiological functions of soybean leaves and roots under saline–alkali stress, the chloroplast and mitochondrial ultrastructure, net photosynthetic rate and root activity of both soybean cultivars were measured after 2 days of NAC treatment. The results showed that in the control group, chloroplasts of both cultivars exhibited a typical spindle shape, with grana and stroma thylakoids arranged in an orderly manner and only a few plastoglobuli (PG) present. Mitochondria appeared oval-shaped, with intact inner and outer membranes and clearly discernible cristae (Figure 7A,D,G,J). Under saline–alkali stress, the chloroplast ultrastructure of both cultivars was severely disrupted: chloroplasts became swollen and deformed, thylakoid arrangement was disordered, the numbers of grana and stroma thylakoids were markedly decreased, and the number of plastoglobuli increased substantially. Mitochondria displayed swelling, abnormal cristae morphology, and irregular outer membrane contours (Figure 7B,E,H,K).

Notably, the extent of mitochondrial damage under saline–alkali stress was greater in HN95 than in HF50. In contrast, exogenous NAC markedly alleviated these ultrastructural impairments: chloroplast morphology became more regular, the stacked thylakoid structure was partially restored, and plastoglobuli accumulation was reduced. Mitochondria retained largely intact ultrastructure, with well-defined cristae and smooth outer membranes, and this restorative effect was more evident in HN95 (Figure 7C,F,I,L). In addition, compared with the control, saline–alkali stress significantly reduced the net photosynthetic rate and root activity of both soybean cultivars, with greater decreases observed in HN95 than in HF50. Exogenous NAC application significantly increased the net photosynthetic rate and root activity of both cultivars under saline–alkali stress, and the improvement in root activity was concentration-dependent (Figure 7M,N).

## 3. Discussion

Saline–alkali stress triggers osmotic stress, ion toxicity, and oxidative stress, which disrupt metabolism, inhibit photosynthate accumulation, and ultimately suppress plant growth [35,36,37]. After 25 days of saline–alkali treatment, shoot and root growth were significantly inhibited in both soybean cultivars, HN95 and HF50 (Figure 1A). Shoot growth inhibition was milder in HN95 than in HF50, whereas roots of HN95 were more severely suppressed under the same stress. Such differential growth responses resulted in an unchanged root-to-shoot ratio in HF50, while that of HN95 decreased markedly.

The root-to-shoot ratio is a key indicator of biomass allocation and stress tolerance in plants subjected to abiotic stress [38]. Previous studies have shown that maintaining a stable root-to-shoot ratio under abiotic stress reflects balanced biomass partitioning between roots and shoots, enabling plants to sustain adequate stress tolerance [39,40]. A decreased root-to-shoot ratio generally indicates more severe stress-induced damage to roots, a response commonly observed in salt-sensitive cultivars [41,42]. Foliar application of NAC at 1 to 3 mmol·L^−1^ significantly alleviated the growth inhibition triggered by saline–alkali stress. NAC application simultaneously promoted shoot and root growth in HF50, producing no significant change in its root-to-shoot ratio. By contrast, NAC more strongly promoted root growth than leaf growth in HN95, thereby increasing its root-to-shoot ratio (Figure 1F).

### 3.1. Exogenous NAC Regulates Cysteine Supply to Restore the hGSH Metabolic Pathway and Rebuild the Antioxidant Redox System

Plant antioxidant enzyme systems and antioxidants effectively mitigate abiotic-stress-triggered oxidative stress in plant cells. As a vital cellular reductant, GSH quenches ROS such as H_2_O_2_ and superoxide anions through reversible redox reactions of its thiol groups, and it also repairs oxidatively damaged enzymes [43]. Moreover, GSH cooperates with the AsA–GSH cycle to confer antioxidative protection to organelles including chloroplasts and mitochondria, thereby stabilizing photosynthetic and respiratory metabolism [44]. As a structural homolog of GSH, hGSH shares similar biological functions. During antioxidation and detoxification, the thiol groups of hGSH are oxidized, and two reduced hGSH molecules conjugate to form oxidized hGSSG. With NADPH serving as the hydrogen donor, hGSH reductase regenerates bioactive hGSH from hGSSG, thereby maintaining cellular redox homeostasis [19].

Upon exposure to abiotic stress, plants upregulate endogenous cysteine biosynthesis to supply precursors for GSH and hGSH production [45]. Cells utilize stored glutamate and cysteine to synthesize hGSH, catalyzed by GCL, with ATP serving as the energy donor (Figure 2A). In the present study, saline–alkali stress markedly elevated cysteine concentrations in roots of the salt-tolerant soybean genotype HF50, whereas cysteine contents in roots and leaves of the salt-sensitive genotype HN95 showed no significant variation (Figure 2B). Meanwhile, saline–alkali stress significantly upregulated the transcript levels of cysteine biosynthetic genes (*GmSERAT1*, *GmSERAT2*, *GmHGS*, *GmGSH1*) in both leaves and roots of HF50, as well as in leaves of HN95, thereby facilitating the biosynthesis of cysteine and hGSH.

Notably, despite the upregulated transcription of these target genes, both soybean cultivars exhibited suppressed GCL activity in roots but enhanced GCL activity in leaves. Such transcriptional–translational uncoupling can be attributed to oxidative inactivation, competition for ATP, and transcriptional feedback inhibition, which jointly reduce GCL catalytic activity in soybean roots under saline–alkali stress [46,47]. In contrast, leaf cells maintain the native conformation of GCL through ion compartmentalization; rapid consumption of (h)GSH further relieves transcriptional feedback inhibition, thereby elevating GCL activity in leaves [48,49]. This tissue-specific uncoupling suggests that soybeans may reduce energy consumption by repressing the synthesis of certain functional enzymes in roots under saline–alkali stress, and shoots may instead provide these enzymes to cooperatively maintain cellular redox homeostasis [50].

NAC is a cysteine prodrug that releases cysteine upon intracellular deacetylation. Earlier studies in mammalian cells have demonstrated that NAC markedly elevates GSH concentrations and enhances antioxidant capacity [51,52]. In the present study, exogenous NAC showed tissue- and genotype-specific effects on cysteine content and GCL activity in the two soybean cultivars. Leaf cysteine concentrations increased with rising NAC concentrations, with a larger increment observed in HN95, implying more efficient NAC uptake or metabolic conversion in this genotype. Conversely, root cysteine levels declined significantly because NAC-induced elevation of GCL activity in both roots and leaves accelerates (h)GSH biosynthesis and rapidly depletes root cysteine pools (Figure 2C). Meanwhile, the long-distance translocation rate of cysteine is inadequate to rapidly replenish root thiol reservoirs [53]. Notably, qRT-PCR data revealed significant downregulation of cysteine biosynthetic genes in leaves and roots of both cultivars, demonstrating that exogenous cysteine supplementation represses endogenous cysteine production. Furthermore, relative to saline–alkali stress alone, exogenous NAC downregulated the expression of genes encoding GCL and hGS in HF50 leaves while upregulating their transcripts in HN95 leaves (Figure 2D). This suggests that NAC-mediated (h)GSH biosynthesis in HF50 relies primarily on post-translational regulation rather than transcriptional activation, including improved substrate utilization efficiency, transcriptional feedback inhibition triggered by high intracellular (h)GSH abundance, and enhanced stability of GCL protein [54,55].

Meanwhile, exogenous NAC increased the activities of AsA–(h)GSH cycle enzymes and other antioxidant enzymes, demonstrating that NAC simultaneously enhanced two functional pathways—regeneration of reductive metabolites and ROS scavenging—thereby jointly alleviating saline–alkali-induced oxidative damage [31,56]. Among the two cultivars, the salt-sensitive HN95 showed greater sensitivity in AsA–(h)GSH-cycle-related indicators (including AsA and (h)GSH concentrations, as well as GR, DHAR and MDHAR activities) to varying NAC concentrations, and the maintenance of cellular redox homeostasis in HN95 depends heavily on thiol precursors supplied by exogenous NAC (Figure 3). Saline–alkali stress significantly reduced the (h)GSH/(h)GSSG and AsA/DHA ratios in roots and leaves, indicating disrupted redox homeostasis under saline–alkali conditions. Exogenous NAC elevated both the (h)GSH/(h)GSSG and AsA/DHA ratios above the saline–alkali stressed level, indicating that NAC restored cellular redox homeostasis under saline–alkali stress (Figure S1).

### 3.2. Exogenous NAC Activates Genome Damage Repair Pathways to Alleviate Oxidative DNA Damage

The alleviation of oxidative damage by NAC led us to ask whether this antioxidant protection was sufficient to preserve genome integrity. Excessive ROS accumulation induced by abiotic stress directly triggers oxidative DNA damage, manifested as the buildup of base oxidation products such as 8-OHdG, accompanied by base mismatches, single- and double-strand breaks, and genomic polymorphisms [57,58]. Our results confirmed that saline–alkali stress significantly induced ROS and 8-OHdG accumulation as well as RAPD polymorphic variation in the roots and leaves of both cultivars, a finding consistent with previous studies [6,8,59]. Under saline–alkali stress, no RAPD polymorphic variations were detected in the leaf genomic DNA of the two soybean cultivars. qRT-PCR results confirmed that saline–alkali stress significantly upregulated the expression of DNA damage repair genes in leaves of HN95 and HF50, thereby enhancing DNA damage repair capacity in leaf tissues (Figure 5). Meanwhile, leaf antioxidant enzymes showed elevated activities and efficiently scavenged ROS, thereby mitigating oxidative damage (Figure 3). These two pathways synergistically sustained leaf genome stability [60,61]. In contrast, roots of both cultivars showed poorer genome stability. On the one hand, saline–alkali stress suppressed the transcript levels of DNA damage repair-related genes in roots, thereby reducing DNA repair efficiency. On the other hand, inadequate antioxidant defenses in roots aggravated oxidative damage (Figure 4).

As a biosynthetic precursor of cysteine, exogenous NAC also possesses intrinsic reductive capacity, acting as an antioxidant and mitigating cellular ROS accumulation. Earlier work demonstrated that NAC seed priming significantly elevated the activities of antioxidant enzymes—including CAT, SOD, POD, APX, and GR—in leaf tissues of *Oryza sativa* L., protected chloroplasts, improved photosynthetic efficiency, and enhanced rice tolerance to transplanting stress [31]. Foliar NAC application increased CAT and SOD activities, elevated endogenous GSH levels and reduced MDA accumulation in leaves of *Solanum nigrum* L., thereby strengthening plant tolerance to cadmium stress [38]. In this study, exogenous NAC upregulated leaf DNA repair pathway genes and boosted antioxidant enzyme activities and antioxidant levels in both soybean cultivars, jointly lowering leaf 8-OHdG accumulation. To extend this assessment within a quantitative integrative framework, NAC treatment substantially increased the rates of recovery from ROS accumulation, MDA accumulation, and 8-OHdG content in seedling roots and leaves of both HF50 and HN95 (Figure S2). In HF50 roots, NAC dose-dependently induced the transcription of DNA damage repair-related genes and reinforced root antioxidant defenses, ultimately improving genome stability. In contrast, NAC treatment exerted no significant effects on the expression of these target genes in HN95 roots, indicating that NAC only alleviates ROS-induced genomic DNA damage by boosting antioxidant defenses and fails to substantially reduce saline–alkali-triggered RAPD polymorphism (Figure 6).

### 3.3. Exogenous NAC Preserves Intact Leaf Ultrastructure and Sustains Photosynthetic Efficiency as Well as Root Activity

Beyond preserving genome integrity, we further investigated whether NAC-mediated protection against oxidative and genomic damage could maintain the structural and functional stability of organelles. Oxidative stress induced by abiotic stresses such as salinity and alkalinity can directly disrupt organellar structures, including those of chloroplasts and mitochondria [62]. Abnormal organellar structures in turn further promote ROS production [63,64]. The maintenance of subcellular structures and physiological homeostasis is fundamental to plant abiotic stress resistance [65]. In the present study, saline–alkali stress caused disordered arrangement of thylakoids and a marked reduction in thylakoid number in soybean chloroplasts; meanwhile, mitochondria were swollen, with damaged cristae and irregular outer membranes, directly impairing photosynthetic carbon assimilation and mitochondrial energy-generating capacity (Figure 7).

Notably, chloroplasts and mitochondria of HN95 exhibited significantly more severe structural damage under saline–alkali stress than those of HF50. In contrast, HF50 accumulated higher levels of leaf ROS, MDA, and 8-OHdG. This discrepancy may arise from genotypic differences in the spatial sites for ROS generation, in situ ROS scavenging capacity, and membrane lipid composition and properties. Electron leakage from the photosynthetic electron transport chain in chloroplasts and the respiratory chain in mitochondria triggers massive local ROS production on membranes, and these ROS production sites differ spatially between the two cultivars [63,66]. The high ROS and MDA levels in HF50 originate predominantly in the apoplast (cell wall and intercellular spaces), generated by plasma membrane NADPH oxidases activated by saline–alkali stress. Given their location, these ROS cause limited direct damage to chloroplasts and mitochondria. In contrast, the ROS burst in HN95 occurs primarily along electron transport chains within organelles, directly attacking thylakoid and inner mitochondrial membranes and triggering structural disintegration.

Furthermore, Cu/Zn-SOD and APX, which are enriched in the chloroplast stroma and around thylakoid membranes, drive in situ ROS scavenging at sites adjacent to organellar membranes [62,64]. The two soybean cultivars likely differ in the subcellular partitioning of intracellular antioxidants, which confers on HF50 superior local ROS scavenging capacity and preserves organellar structural integrity. Variations between the two cultivars in the proportion and content of saturated fatty acids and sterols in chloroplast and mitochondrial membranes further altered the physical rigidity of lipid bilayers and their ability to block lipid peroxidation chain reactions. The salt–alkali-tolerant genotype HF50 possesses a membrane skeleton with greater resistance to collapse. Even with elevated MDA concentrations, it effectively restricted the spread of LPO chain reactions toward membrane protein complexes and retained intact membrane morphology [19,20,38]. Collectively, saline–alkali stress induced more severe organellar damage and impaired organellar functions in HN95. This suggests that, when assessing plant stress tolerance, evaluating the physical structural retention capacity of organelles may offer more valuable indicators than measuring total ROS accumulation and scavenging levels. As expected, NAC treatment markedly improved leaf structural stability of both soybean cultivars under saline–alkali stress, sustained high photosynthetic capacity and root activity, and ultimately enhanced plant tolerance to saline–alkali stress. Unlike previous reports [31,38], where NAC primarily acts by elevating antioxidant enzyme activities or GSH content, our findings reveal that, in soybean under saline–alkali stress, NAC coordinates hGSH metabolism, DNA repair, and organelle protection in a genotype-dependent manner, highlighting novel regulatory features of NAC action in soybean.

## 4. Materials and Methods

### 4.1. Materials, Growth, and Treatment Conditions

Two soybean cultivars differing in saline–alkali tolerance—Hefeng 50 (HF50, tolerant) and Henong 95 (HN95, sensitive)—were used as experimental materials, with seeds provided by the Innovative Team for Stress-Resistant Legume Cultivation, Heilongjiang Bayi Agricultural University. Soybean seedlings were cultivated in a controlled-environment growth room under a 16 h light/8 h dark photoperiod, at a constant temperature of 25 ± 2 °C, 50–55% relative humidity, and a photosynthetic photon flux density of 600 μmol·m^−2^·s^−1^. A pot culture experiment was performed using a peat soil: vermiculite substrate (3:1, *v*/*v*), with 1.5 kg of the mixture filled into each plastic pot. Plump, undamaged seeds were selected for sowing, and half-strength Hoagland’s nutrient solution was supplied at regular intervals. After emergence, three uniform seedlings were retained per pot and defined as one biological replicate, with nine replicates per treatment arranged in a completely randomized design. Leachate drained from each pot was returned via a drainage tray to its original pot to maintain uniform water and salt conditions across all substrates.

At the V1 growth stage (17 days after sowing), combined saline–alkali stress and foliar NAC (Cat. C8460, Solarbio Life Sciences, Beijing, China) application was initiated simultaneously. A total volume of 1.5 L saline–alkali nutrient solution containing 4 mmol·L^−1^ NaCl, 4 mmol·L^−1^ Na_2_CO_3_, 36 mmol·L^−1^ NaHCO_3_ and 36 mmol·L^−1^ Na_2_SO_4_, all purchased from Liaoning Quanrui Reagent Co., Ltd. (Jinzhou, China), prepared at pH 9.8 and irrigated into each pot over three consecutive days. Concurrently, NAC solution was applied via foliar spraying at 6 mL per seedling once daily for three successive days, with 0.01% Silwet-77 (Yisheng Biotechnology Co., Ltd., Guangzhou, China) added to all spray solutions as a surfactant. Five experimental treatments were established: CK (normal nutrient solution + distilled water foliar spray), SA (saline–alkali stress + distilled water foliar spray), N1, N2 and N3 (saline–alkali stress combined with foliar spraying of 1, 2 and 3 mmol·L^−1^ NAC, respectively). The 1 mmol·L^−1^ NAC concentration was chosen based on our preliminary experiments and previous research demonstrating its capacity to alleviate saline–alkali-stress-induced growth suppression in soybean seedlings [34]. Higher concentrations (2 and 3 mmol·L^−1^) were further included to explore possible dose-dependent responses to NAC application.

Two sampling stages were arranged for subsequent analyses. In the first stage, seedlings were cultured continuously after the completion of foliar spraying, with NAC re-applied every 7 days, until pronounced phenotypic differences became apparent among treatments; morphological traits and dry biomass accumulation were then measured to evaluate the alleviation effect of NAC on saline–alkali-induced growth inhibition. In the second stage, two days after termination of foliar spraying, the first trifoliate leaves and root systems of soybean plants were harvested, snap-frozen in liquid nitrogen, and stored at −80 °C for subsequent physiological and molecular biomarker assays, intended to elucidate the regulatory pathways and molecular mechanisms through which NAC modulates saline–alkali tolerance in soybean. Each assay included three biological replicates, each with three technical replicates.

### 4.2. Determination of Phenotypic Traits and Dry Matter

Plant height of soybean was measured with a ruler, from the cotyledonary node to the apical meristem of the stem. Total leaf area of soybean seedlings was determined using a leaf area meter (Yaxin-1241, Beijing Yaxinliyi Science and Technology, Beijing, China). Root system images were acquired using a root scanner (Phantom 9900XL, Microtek Shanghai Technology Co., Ltd., Shanghai, China), and various root morphological parameters were analyzed using WinRHIZO software (Pro 2016a, Regent Instruments Inc., Quebec City, QC, Canada). Fresh plant tissues were first oven-dried in a drying oven (BGZ-246, Shanghai Boxun Medical Biological Instrument Corp., Shanghai, China) at 105 °C for 30 min to inactivate enzymes, then transferred to 80 °C and dried to constant weight; dry weight was subsequently recorded. Each assay contained three biological replicates, each comprising three technical replicates.

### 4.3. Histochemical Staining of O_2_^−^ and H_2_O_2_

Histochemical staining of superoxide anion (O_2_^−^) and hydrogen peroxide (H_2_O_2_) was performed on the first trifoliate leaves and roots of soybean seedlings using NBT (Cat. N814596, Shanghai Macklin Biochemical Co., Ltd., Shanghai, China) and DAB (Cat. D821272, Shanghai Macklin Biochemical Co., Ltd., Shanghai, China), respectively. For O_2_^−^ detection, samples were immersed in 50 mmol·L^−1^ sodium phosphate buffer (pH 7.0) containing 0.5 mg·mL^−1^ NBT; for H_2_O_2_ detection, samples were immersed in 10 mmol·L^−1^ Tris-HCl buffer (pH 6.5) supplemented with 1.0 mg·mL^−1^ DAB. Stained samples were incubated in darkness at 25 °C for 12–16 h; leaves were heated in decolorizing solution (absolute ethanol: glacial acetic acid = 3:1, *v*/*v*) at 70 °C to eliminate chlorophyll interference; and images were captured once color development was clearly visible [67]. Reactive oxygen species accumulation was evaluated based on the intensity of blue precipitates for O_2_^−^ and brown precipitates for H_2_O_2_.

### 4.4. Determination of ROS, LPO, and MDA Contents

Approximately 0.2 g of frozen samples (stored at −80 °C) were weighed and processed to prepare extraction solutions for the measurement of O_2_^−^, H_2_O_2_, LPO, and MDA. The content of O_2_^−^ in tissues was determined using the hydroxylamine hydrochloride method, and H_2_O_2_ content was measured by the titanium sulfate method, both following the protocol described by Shi [68]. LPO and MDA contents were quantified by the thiobarbituric acid (TBA)-reactive substances method based on Hodges [69], employing a Solarbio Lipid Peroxidation Assay Kit (Cat. BC5240, Solarbio Science & Technology Co., Ltd., Beijing, China) and a Malondialdehyde Assay Kit (Cat. BC0025, Solarbio Science & Technology Co., Ltd., Beijing, China) for LPO and MDA quantification, respectively. Each assay included three biological replicates, each with three technical replicates.

### 4.5. Determination of Cysteine Content and GCL Activity

Approximately 0.2 g of frozen samples (stored at −80 °C) was weighed and processed to prepare extraction solutions for the determination of cysteine content and GCL activity. Cysteine content was quantified using the phosphomolybdic acid reduction method [70], employing a Grace Biotech Cysteine Assay Kit (Cat. G0428, Grace Biotech Co., Ltd., Suzhou, China). GCL activity was assayed by measuring the rate of inorganic phosphate released from the γ-glutamylcysteine biosynthetic reaction as described by May and Leaver [71], employing a Solarbio GCL Activity Assay Kit (Cat. BC1215, Solarbio Science & Technology Co., Ltd., Beijing, China), with minor modifications. Each assay included three biological replicates, each with three technical replicates.

### 4.6. Determination of (h)GSH, (h)GSSG, AsA, and DHA Contents

Approximately 0.2 g aliquots of samples (stored at −80 °C) were individually homogenized to prepare extraction solutions for the quantification of multiple antioxidant indicators. The combined reduced thiol pool—comprising GSH and hGSH (collectively (h)GSH), the combined oxidized thiol pool (GSSG and hGSSG, denoted (h)GSSG), reduced ascorbate, and total ascorbate (total AsA)—were quantified using three colorimetric assay kits (Cat. BC1175, BC1180, BC4635, Solarbio Science & Technology Co., Ltd., Beijing, China). Specifically, (h)GSH was measured by an Ellman reaction with DTNB at 412 nm, while (h)GSSG was measured by a GR/NADPH recycling assay with 2-vinylpyridine masking of endogenous GSH [68,72]. Reduced AsA and total AsA were determined via the Fe^3+^ reduction/2,2′-bipyridine colorimetric method; DHA concentrations were calculated by subtracting reduced AsA from total AsA [73,74]. Because the commercial kits employed in this study cannot discriminate between GSH and hGSH, nor between GSSG and hGSSG, the abbreviations (h)GSH and (h)GSSG are used consistently throughout to denote the total reduced and total oxidized thiol fractions, respectively. All measurements were performed with three independent biological replicates, each containing three technical replicates.

### 4.7. Determination of GR, DHAR, MDHAR, SOD, CAT, POD, and APX Activities

Approximately 0.2 g of frozen samples (stored at −80 °C) was weighed and processed to prepare extracts for the determination of different physiological indicators. The activities of GR, DHAR, MDHAR, SOD, CAT, POD, and APX were assayed using commercial kits (Cat. BC1160, BC0665, BC0650, BC0175, BC0200, BC0095, BC0225; Solarbio Science & Technology Co., Ltd., Beijing, China), with all extraction and reaction procedures conducted on ice to maintain enzyme activity.

Enzymatic activities were quantified using a microplate reader (SpectraMax 190, Molecular Devices, Sunnyvale, CA, USA). Specifically, GR activity was determined by monitoring NADPH oxidation at 340 nm [68]; MDHAR activity by monitoring NADH oxidation at 340 nm [75]; DHAR activity by monitoring DTNB color development at 412 nm [76]; SOD activity by the NBT photoreduction inhibition method at 560 nm, with 1 U defined as the enzyme amount causing 50% inhibition of NBT reduction [68]; CAT activity by monitoring H_2_O_2_ decomposition at 240 nm [68]; POD activity by monitoring guaiacol oxidation at 470 nm [68]; and APX activity by monitoring ascorbate oxidation at 290 nm [68]. Each treatment contained three independent biological replicates, with each biological replicate comprising three technical replicates.

### 4.8. Determination of RAPD Profiles and Genomic Stability

Genomic DNA was extracted from samples stored at −80 °C using a Plant Genomic DNA Extraction Kit (Cat. 4992201, Tiangen Biotech Co., Ltd., Beijing, China). RAPD polymorphism analysis was performed according to Wang et al. using random primers 2 and 9, which were synthesized by Sangon Biotech (Shanghai) Co., Ltd. (Shanghai, China), with the following 5′→3′ sequences: TCCGATGCTG and AAAGTGCGGC [6,77]. Genomic stability was calculated following Zhao [78]. All experiments were conducted with three biological replicates, each comprising three technical replicates.

### 4.9. Determination of 8-OHdG Content

The 8-OHdG content in the samples was determined using an 8-OHdG ELISA kit (Cat. JRX914286, Quanzhou Ruixin Biotechnology Co., Ltd., Quanzhou, Fujian, China). For 8-OHdG measurement, 0.1 g of tissue samples stored at −80 °C was ground in liquid nitrogen and homogenized in 900 μL of ice-cold phosphate-buffered saline (PBS; 0.01 mol·L^−1^, pH 7.4). The homogenates were centrifuged at 5000× *g* for 10 min, and the supernatants were collected for analysis. According to the manufacturer’s instructions, 90 μL of 1× assay buffer and 10 μL of sample supernatant were added to each well, followed by 50 μL of detection antibody (diluted 1: 100). The plate was incubated at room temperature for 1.5 h, washed six times, and then incubated with 100 μL of streptavidin–HRP conjugate (diluted 1: 100) at room temperature in the dark for 30 min. After six additional washes, 100 μL of chromogenic substrate was added and allowed to react at room temperature in the dark for 15 min. The reaction was terminated by adding 100 μL of stop solution, and the absorbance was measured at 450 nm. The 8-OHdG concentration was calculated based on a standard curve generated in parallel. Each assay included three biological replicates, each comprising three technical replicates.

### 4.10. Determination of Leaf Microstructure

Fresh leaf samples (1 cm × 1 cm) were immersed in electron microscopy fixative solution (Cat. G1102, Wuhan Servicebio Technology Co., Ltd., Wuhan, Hubei, China). Ultrastructural analysis was performed using transmission electron microscopy (HT7700, Hitachi, Tokyo, Japan) according to the protocol described by Zhao [8].

### 4.11. Measurement of Net Photosynthetic Rate

The net photosynthetic rate was determined on the first fully expanded trifoliate leaf of soybean seedlings using a portable photosynthesis system (LI-6800, LI-COR Biosciences, Lincoln, NE, USA). The leaf chamber parameters were set as follows: airflow rate of 600 μmol·s^−1^, reference CO_2_ concentration of 400 μmol·mol^−1^, relative humidity of 65%, mixing fan speed of 10,000 rpm, leaf temperature of 25 °C, and photosynthetically active radiation (PAR) of 1500 μmol·m^−2^·s^−1^. Data were recorded after all photosynthetic parameters stabilized. All measurements included three independent biological replicates, each comprising three technical replicates.

### 4.12. Determination of Root Activity

Root viability was determined following Gao’s protocol via 2,3,5-triphenyltetrazolium chloride (TTC, Cat. T8170, Solarbio Science & Technology Co., Ltd., Beijing, China) assay [74]. Fresh root tip samples (0.1 g) were immersed in 10 mL mixed solution containing equal volumes of 1% TTC solution and 0.1 mol·L^−1^ phosphate buffer. The samples were incubated at 37 °C for 1 h in darkness, and the reaction was terminated by adding 2 mL of 1 mol·L^−1^ H_2_SO_4_. Triphenyl formazan produced in root tips was extracted with ethyl acetate, and the absorbance was determined at 485 nm. TTC reduction intensity, expressed per unit fresh weight, was calculated as the indicator of root viability. All measurements included three independent biological replicates, each comprising three technical replicates.

### 4.13. RNA Extraction, First-Strand cDNA Synthesis, and qRT-PCR Analysis

Total RNA was extracted from soybean tissues using a TransZol Plant Kit (Cat. ET121, TransGen Biotech Co., Ltd., Beijing, China). RNA concentration was quantified using a NanoDrop One spectrophotometer (ND-ONE, Thermo Fisher Scientific, Waltham, MA, USA), and RNA integrity was assessed by 1% agarose gel electrophoresis. RNA was reverse-transcribed into cDNA using HiScript III RT SuperMix for qPCR (+gDNA wiper) (Cat. R323-01, Vazyme Biotech Co., Ltd., Nanjing, China). qRT-PCR was performed on a Q9600 Series Real-Time PCR System (Q9606, Bio-Gener Technology Co., Ltd., Hangzhou, China) using diluted cDNA products with ChamQ Universal SYBR qPCR Master Mix (Cat. Q711-02, Vazyme Biotech Co., Ltd., Nanjing, China). Relative gene expression was calculated using the 2^−ΔΔCt^ method with *GmEF1a* as the reference gene. Primer sequences are listed in Table S1. The expression level of the control group was defined as 1. Each treatment included three independent biological replicates, each comprising three technical replicates.

### 4.14. Statistical Analysis

Statistical analyses were performed using IBM SPSS Statistics (version 19.0; IBM Corp., Armonk, NY, USA). Data are presented as mean ± SD from three independent experiments. One-way analysis of variance was performed separately for each genotype to evaluate the dose-dependent effects of NAC treatments. Fisher’s Least Significant Difference post hoc test was used for multiple comparisons. A *p*-value < 0.05 was considered statistically significant. Graphical illustrations were generated using OriginPro 2025 (OriginLab Corporation, Northampton, MA, USA).

## 5. Conclusions

This study reveals that exogenous foliar application of NAC can effectively reduce saline–alkali-stress-induced ROS accumulation and oxidative damage in soybean plants, and enhance plant tolerance to saline–alkali stress. Foliar spraying of NAC effectively supplies cysteine substrates to plants, activates the AsA–(h)GSH cycle, and enhances antioxidant defense capacity. Meanwhile, it activates the nuclear DNA damage repair pathway, markedly reduces oxidative and genomic damage in seedlings, and preserves the structural integrity and functional stability of chloroplasts and mitochondria. Furthermore, soybean exhibits distinct genotypic and tissue-specific responses to NAC treatment. The stress alleviation effect of NAC on HN95 is concentration-dependent, and its roots mainly resist saline–alkali injury by boosting antioxidant metabolism. In contrast, HF50 coordinates antioxidant metabolism and DNA damage repair capacity, showing greater adaptability to varying NAC concentrations. Although this study establishes a multi-dimensional protective role of NAC at the physiological and molecular levels, the upstream signaling pathways through which NAC activates DNA repair, as well as the field efficacy and long-term agronomic performance of NAC application, remain to be further investigated before field translation.

## Data Availability

The original contributions presented in this study are included in the article/Supplementary Material. Further inquiries can be directed to the corresponding authors.

## Supplementary Materials

The following supporting information can be downloaded at: https://www.mdpi.com/article/10.3390/plants15162445/s1, Table S1: Primers used for qRT-PCR analysis; Figure S1: Effects of exogenous NAC on (h)GSH/(h)GSSG and AsA/DHA ratios in roots and leaves of soybean seedlings under saline–alkali stress. (A) (h)GSH/(h)GSSG ratio. (B) AsA/DHA ratio. Ratios were calculated from the mean content values of (h)GSH, (h)GSSG, AsA and DHA reported in Figure 2. Data are presented without error bars because they were derived from treatment group means rather than within-replicate values. Figure S2: Effects of exogenous NAC on oxidative damage recovery rates in leaves and roots of soybean seedlings under saline–alkali stress. Heatmap colors represent oxidative damage recovery rates calculated from the measured contents of O_2_^−^, H_2_O_2_, LPO, MDA and 8-OHdG. The recovery rate was calculated as: recovery rate(%) = (*I*_SA_ − *I*_N_)/(*I*_SA_ − *I*_CK_) × 100%. *I*_CK_, measured value under control treatment; *I*_SA_, measured value under saline–alkali stress; *I*_N_, measured value under saline–alkali stress plus exogenous NAC.

## Abbreviations

The following abbreviations are used in this manuscript:

8-OHdG	8-hydroxy-2′-deoxyguanosine
APX	Ascorbate peroxidase
AsA	Ascorbic acid
BER	Base excision repair
CAT	Catalase
DAB	3,3′-Diaminobenzidine
DHA	Dehydroascorbic acid
DHAR	Dehydroascorbate reductase
GCL	Glutamate–cysteine ligase
GR	Glutathione reductase
GSH	Glutathione
GSSG	Oxidized glutathione
hGSH	Homoglutathione
hGSSG	Oxidized homoglutathione
HR	Homologous recombination
LPO	Lipid peroxidation
MDA	Malondialdehyde
MDHA	Monodehydroascorbate
MDHAR	Monodehydroascorbate reductase
MMR	Mismatch repair
NAC	N-acetyl-L-cysteine
NBT	Nitro-blue tetrazolium
NER	Nucleotide excision repair
NHEJ	Non-homologous end-joining
PAR	Photosynthetically active radiation
POD	Peroxidase
RAPD	Random amplified polymorphic DNA
RBOH	NADPH oxidase
RNA	Ribonucleic acid
ROS	Reactive oxygen species
R/S	Root-to-shoot ratio
SOD	Superoxide dismutase
TEM	Transmission electron microscopy
