# Supplementary material for: Exogenous N-Acetyl-L-cysteine Improves Soybean Saline–Alkali Tolerance by Enhancing the hGSH Pathway to Alleviate Oxidative Damage
Source: Plants (Basel). 2026 Aug 11;15(16):2445. doi: 10.3390/plants15162445 (PMC13516288; doi:10.3390/plants15162445)
Supplement: Supplementary file 1 [file plants-15-02445-s001.zip › plants-4450652-supplementary.pdf]

Table S1 Primers used for qRT-PCR analysis

Gene name	Gene ID	Forward primer (5'-3')	Reverse primer (5'-3')
GmSERAT1	GLYMA14G014700	TTGGTGCTGGTTCTGTGGTT	AAGTCAGCAATATGTGAAGTATGG
GmSERAT2	GLYMA11G084100	ATGACGACGCCGAACCTATC	GACGATTAGGGGGGCACAAAC
GmGSH1	GLYMA05G207600	ATTCCTGAACCCTAAGCGCC	CTACGGCCACCAACAATCCT
GmHGS	GLYMA19G237700	GTGCACTTCTAACAACACCACC	GCTCCAGACAAGAGCATCGT
GmNBS1	GLYMA14G208100	TGACTGTGGGAATGAGGACGTG	AGAACCAACAGTGCCTCGTGAAC
GmBRCA1	GLYMA19G166900	CTGCATGCTTCCTCCATGTTGC	TAAGCAGCCCGACTTTGGCTTG
GmRAD51	GLYMA11G143900	AGTAGATGGTTCTGCAGTCTTTGC	CTTTCCTGAGAGCTAGCCTCGTTG
GmMRE11	GLYMA12G219100	TGGGCACTGACGAAAGCAAACC	GCATAACACAACCTGGCTGCAATG
GmKU70	GLYMA20G248800	TCGAGGCGTTTATTGGGAGGAAAG	TTTCCGGTGAGATTGGCTGGTTG
GmKU80	GLYMA05G125700	CTGGAATGTTTAGTTGCACTTCGC	ATCATTGAACTGTTCCGGTTCCTG
GmMLH1	GLYMA04G254900	TGAAGAGAATACCTGTGATAATAC	TCCATCAGTAGCCATAGAAG
GmMSH2	GLYMA17G121400	TCATCGGACGAAGGAGATATTACT	CACCCACCTGTCGGATAAATG
GmMSH6	GLYMA19G216000	ATCCGAAGGTCTGTCTCT	AGCAACATTAACACCATAGC
GmMMH-2	GLYMA13G366800	TGATCTGTTGGTCGGCTTGTGC	AGGCATCCTACTCTTGCCTTCC
GmOGG1	GLYMA06G115500	TGGGTCCTAAAGTGGCTGCTTG	ACGTGTGTGTCAACAGGAACAGC
GmNTH1	GLYMA11G128600	TCAAGCCCATCTTCTTCCAAGTCC	TGTCCTTGTCCATGTCAAACCC
GmNTH2	GLYMA13G343700	GCAATCGTCTTGGATGGGTTTCG	GCAGCGCTTGTCTTGTTTCTTCC
GmCSA1	GLYMA04G045700	CGCTGATGAAATGGAAGGAACTGC	TGTCACACAAAGGGAGGCAAGAC
GmRAD4	GLYMA14G214000	ATGGGAAGTGGCAACTGGAACC	ACTTGACCTCGCTCGTTCTTGG

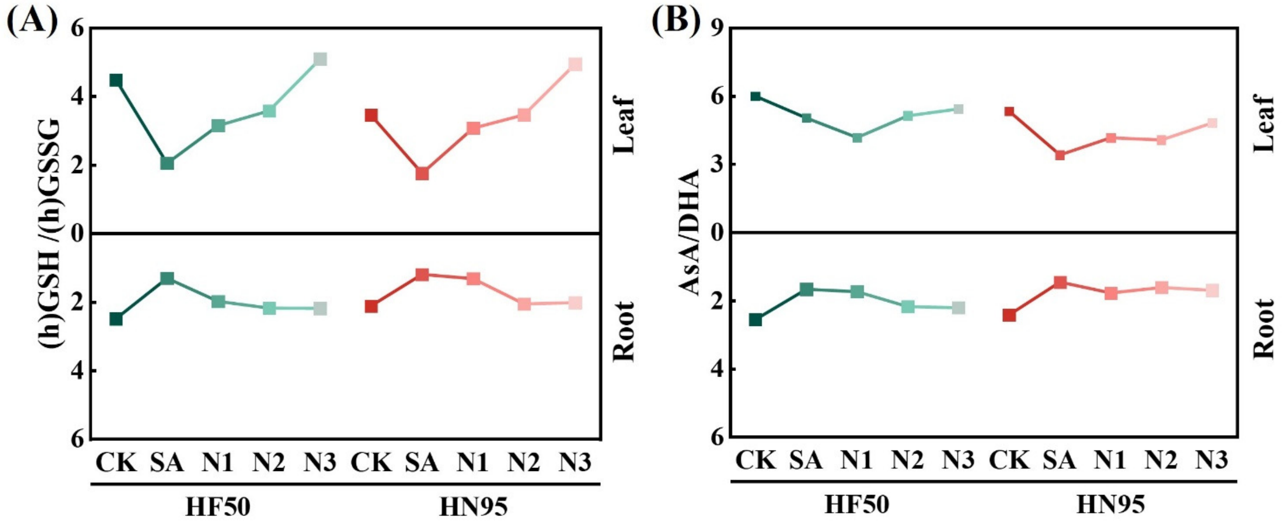

Figure S1. Effects of exogenous NAC on (h)GSH/(h)GSSG and AsA/DHA ratios in roots and leaves of soybean seedlings under saline-alkali stress. (A) (h)GSH/(h)GSSG ratio. (B) AsA/DHA ratio. Ratios were calculated from the mean content values of (h)GSH, (h)GSSG, AsA and DHA reported in Figure 2. Data are presented without error bars because they were derived from treatment group means rather than within-replicate values.

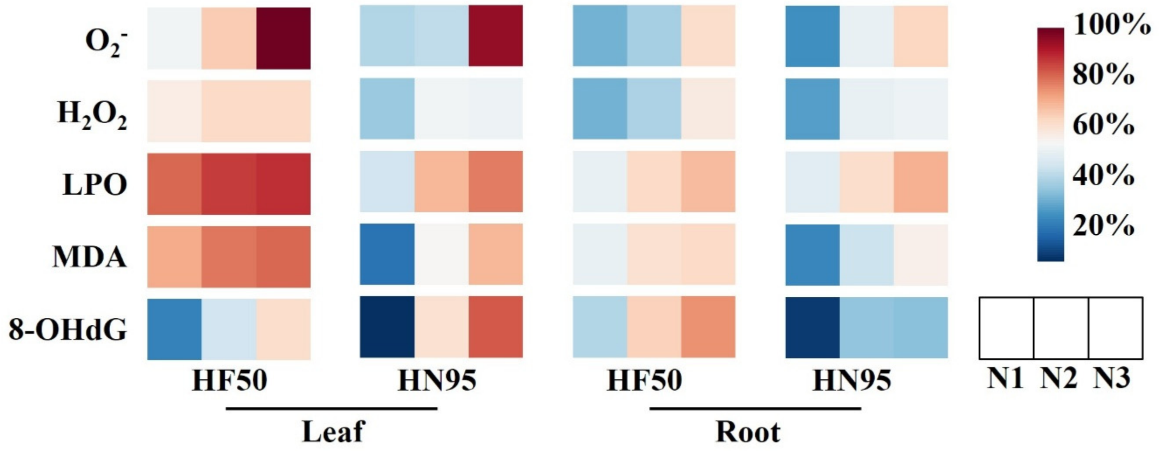

Figure S2. Effects of exogenous NAC on oxidative damage recovery rates in leaves and roots of soybean seedlings under saline-alkali stress. Heatmap colors represent oxidative damage recovery rates calculated from the measured contents of O_2^-, H_2O_2, LPO, MDA and 8-OHdG. The recovery rate was calculated as: Recovery rate(%) = $(I_{SA}-I_N)/(I_{SA}-I_{CK})\times 100\%$. I_{CK}, measured value under control treatment; I_{SA}, measured value under saline-alkali stress; I_N, measured value under saline-alkali stress plus exogenous NAC.
